# Identification in the mould *Hypocrea jecorina* of a gene encoding an NADP^+^: d-xylose dehydrogenase

**DOI:** 10.1111/j.1574-6968.2007.00969.x

**Published:** 2007-11

**Authors:** Suvi Berghäll, Satu Hilditch, Merja Penttilä, Peter Richard

**Affiliations:** VTT Technical Research Centre of Finland Espoo, Finland

**Keywords:** d-xylose dehydrogenase, d-xylose metabolism, *Hypocrea jecorina*, *Trichoderma reesei*, NADP-linked d-xylose dehydrogenase, EC 1.1.1.175

## Abstract

A gene coding for an NADP^+^-dependent d-xylose dehydrogenase was identified in the mould *Hypocrea jecorina* (*Trichoderma reesei*). It was cloned from cDNA, the active enzyme was expressed in yeast and a histidine-tagged enzyme was purified and characterized. The enzyme had highest activity with d-xylose and significantly smaller activities with other aldose sugars. The enzyme is specific for NADP^+^. The *K*_m_ values for d-xylose and NADP^+^ are 43 mM and 250 μM, respectively. The role of this enzyme in *H. jecorina* is unclear because in this organism d-xylose is predominantly catabolized through a path with xylitol and d-xylulose as intermediates and the mould is unable to grow on d-xylonic acid.

## Introduction

Yeasts and moulds ferment d-xylose through an oxidoreductive pathway where d-xylose is first reduced to xylitol by a d-xylose reductase (Chiang & Knights, 1959). This reductase is either specific or has a preference for NADPH as a cofactor (Verduyn *et al.*, 1985). In the second step xylitol is oxidized to d-xylulose by an NAD^+^-specific xylitol dehydrogenase. An exception is the anaerobic fungus *Piromyces* sp. strain E2 which has a d-xylose isomerase to convert d-xylose to d-xylulose directly (Harhangi *et al.*, 2003). In prokaryotes the path through the d-xylose isomerase is predominant.

There are at least two other pathways for the catabolism of d-xylose which were described only in prokaryotes (Weimberg, 1961; Dahms, 1974) (see also Stephens *et al.*, 2007). Common in these two pathways is that d-xylose is first oxidized by a d-xylose dehydrogenase. The d-xylose dehydrogenase oxidizes d-xylose to d-xylono-1,4-lactone utilizing NAD^+^ or NADP^+^ as a cofactor. The d-xylono-1,4-lactone is then hydrolysed to d-xylonic acid. In the next step a dehydratase converts d-xylonic acid to 3-deoxy-d-glycero-pentulosonic acid (2-keto-3-deoxy-d-xylonic acid). For the further conversion of 3-deoxy-d-glycero-pentulosonic acid two different paths are possible. In the first path an aldolase splits 3-deoxy-d-glycero-pentulosonic acid into glycoaldehyde and pyruvic acid (Dahms, 1974). In the second path another dehydratase converts 3-deoxy-d-glycero-pentulosonic acid to α-ketoglutaric semialdehyde, which is subsequently oxidized by an NAD^+^-utilizing dehydrogenase to α-ketoglutarate (Weimberg, 1961). Similar prokaryotic paths were described for l-arabinose (Watanabe *et al.*, 2006a, 2006b).

A fungal catabolic pathway where d-xylose is first oxidized and then further metabolized has not been described. However, there are reports about d-xylose dehydrogenase activities in eukaryotic microorganisms. In the mould *Trichoderma viride* a d-xylose dehydrogenase activity was reported (Kanauchi & Bamforth, 2003). This enzyme used NAD^+^ as a cofactor and was active with the sugars d-glucose, d-xylose and l-arabinose. The *K*_m_ values for these sugars were 220, 800 and 1450 mM, respectively. It was suggested that the enzyme was primarily a d-glucose dehydrogenase. A fungal NADP^+^-dependent d-xylose dehydrogenase activity had been described in the yeast *Pichia quercuum* (Suzuki & Onishi, 1973). This yeast species had also NADPH-coupled d-xylose reductase activity which complicated the assaying of the d-xylose dehydrogenase because the NADPH formed by the dehydrogenase was immediately consumed by the reductase. The authors were able to assay the dehydrogenase activity only after partial purification of the enzyme.

Also in higher eukaryotes an NADP^+^-dependent d-xylose dehydrogenase has been described; it was purified from pig liver (Zepeda *et al.*, 1990). The d-xylose dehydrogenase was identical with the dimeric dihydrodiol dehydrogenase for which gene sequences were known (Aoki *et al.*, 2001). Although fungal d-xylose dehydrogenase activities have been described, the corresponding gene sequences have not been reported.

## Materials and methods

### Strains and growth conditions

The *Hypocrea jecorina* strain VTT-D-80133 was tested for growth with 40 g L^−1^ magnesium d-xylonate as described earlier (Kuorelahti *et al.*, 2006). Magnesium d-xylonate was prepared by oxidation of d-xylose with periodic acid as described (Moore & Link, 1940).

### Cloning of the d-xylose dehydrogenase gene

The d-xylose dehydrogenase gene was cloned by PCR using a *H. jecorina* cDNA library (Margolles-Clark *et al.*, 1996) as a template. The following primers were used in sense direction: 5′-AGATCTACCATGGCGTCTGGAAACCCTT-3′ and in antisense direction: 5′-AGATCTTCTACTGATTCCCCGTGTTGA-3′, each of which contained a BglII restriction site (underlined). The PCR product was then cloned into the pCR2.1 TOPO vector (Invitrogen) and sequenced. To clone the ORF coding for C-terminally histidine-tagged enzyme the same procedure was used except that the following primer was used for the antisense direction: 5′-AGATCTCTAGTGATGGTGATGGTGATGCTGATTCCCCGTGTTGAGA-3′. The BglII fragments with the ORFs were then cloned in the corresponding site of the yeast expression vector p1181 (Verho *et al.*, 2003) which contained the *PGK1* promoter and the *URA3* gene for selection.

### Expression in yeast

The yeast expression vectors were transformed to the *Saccharomyces cerevisiae* strain CEN-PK2-1D (VW-1B) and grown on selective medium lacking uracil with d-glucose as a carbon source. A control strain contained the empty expression vector p1181. To obtain cell extracts the yeast cells were harvested by centrifugation and washed once with water. Approximately equal volumes of cell cake, buffer [10 mM sodium phosphate pH 7.0 containing protease inhibitor (Complete, Roche)] and glass beads with a diameter of 0.5 mm were vortexed in a Mini Bead Beater (Stratec Scientific) two times for 1 min. The mixture was then centrifuged for 15 min at 16 000 ***g*** and the supernatant analysed. For the purification of the histidine-tagged enzyme this crude extract was applied to a nickel/nitrilotriacetic acid column (Qiagen) and eluted according to the manufacturer's instructions.

### Enzyme assays

The standard assay to measure the d-xylose dehydrogenase activity was made in a buffer containing 100 mM Tris/HCl pH 8.1, 2 mM MgCl_2_ and 1 mM NADP^+^ and 100 mM d-xylose. Upon addition of the enzyme preparation the NADPH absorbance was monitored at a wavelength of 340 nm. The assays were performed at 30 C in a Cobas Mira+ automated analyser.

### Product identification

The reaction product was identified by HPLC using a Dionex DX 500 system equipped with a Dionex CarboPac PA-1 column. The column was eluted with a gradient from 0 to 300 mM sodium acetate in 100 mM NaOH. Calcium d-xylonate was prepared by oxidation of d-xylose with periodic acid (Moore & Link, 1940) and used as a standard.

## Results

The *H. jecorina* genome sequence was searched (http://genome.jgi-psf.org/) for sequences with homologies to a d-xylose dehydrogenase earlier described for the halophilic archaeon *Haloarcula marismortui* (Johnsen & Schönheit, 2004) and found six potential genes with homologies. These homologous sequences were used to design primers which were used to try to amplify the homologous DNA sequences from a cDNA library (Margolles-Clark *et al.*, 1996) by PCR. For one of the six homologous sequences a PCR product was obtained. The complete ORF was then obtained by PCR using one primer from the cDNA and another from the cDNA library vector as described before (Richard *et al.*, 2001). An ORF with 1173 bases was obtained. The cDNA sequence of the ORF was deposited in GenBank (http://www.ncbi.nlm.nih.gov/Genbank/) and has the accession number EF136590. The ORF codes for a protein with 391 amino acids and a calculated molecular mass of 42.720 Da. Comparison of the cDNA with the genomic DNA revealed two introns in the genome sequence. They were after nucleotides 64 and 501 of the ORF and contained 68 and 60 nucleotides, respectively. In this study the gene was called *xyd1*, for d-xylose dehydrogenase.

The C-terminal end of the protein showed homologies to proteins of the GFO-IDH-MocA oxidoreductase protein family. This part of the protein contains the NAD(P)^+^ Rossmann-fold, the nucleotide binding site. The protein shows also homologies to the MviM protein family of predicted dehydrogenases and related proteins.

The *xyd1* of *H. jecorina* had highest homology to a group of proteins from filamentous fungi which were all hypothetical proteins of unknown function. The *xyd1* exhibited also some similarity (about 30% identities in the amino acid sequence) to the dihydrodiol dehydrogenase from pig liver which has also d-xylose dehydrogenase activity (Aoki *et al.*, 2001).

**Fig. 1:**
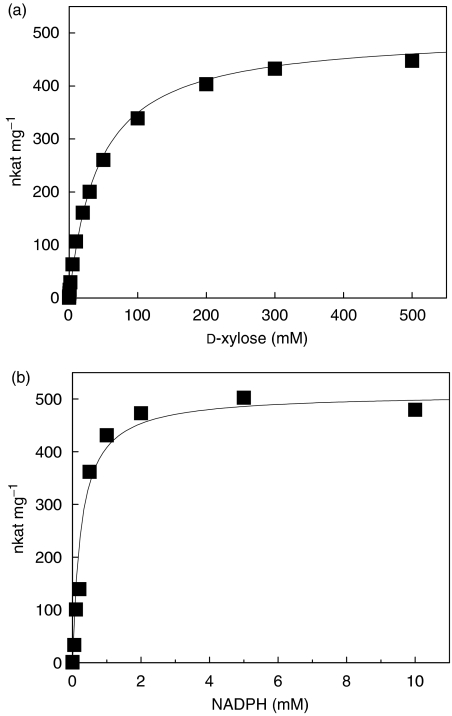
Kinetics of the C-terminally histidine-tagged and purified d-xylose dehydrogenase. (a) The concentration of NADP^+^ was 0.8 mM. (b) The concentration of d-xylose was 300 mM. The curves are calculated Michaelis–Menten kinetics using the constants as indicated. For d-xylose, *K*_m_ 43 mM and *V*_max_ 500 nkat mg^−1^ and for NADP^+^, *K*_m_ 250 μM and *V*_max_ 510 nkat mg^−1^ were used.

The gene was expressed in the yeast *S. cerevisiae* under the strong and constitutive *PGK1* promoter from a multicopy plasmid. The crude cell extract showed an activity with NADP^+^ and d-xylose of about 14 nkat (nmol s^−1^) mg^−1^ of extracted protein. The actual activity might be even higher because it is known that *S. cerevisiae* has some NADPH coupled d-xylose reductase activity (Kuhn *et al.*, 1995). The reductase would consume the NADPH produced by the dehydrogenase. Lower dehydrogenase activity was observed with d-glucose, 4 nkat mg^−1^, d-galactose, 2 nkat mg^−1^, and l-arabinose, 2 nkat mg^−1^. The extract of the control strain that contained the empty plasmid did not show activity (<0.1 nkat mg^−1^) with any of the sugars. Replacing the NADP^+^ by NAD^+^ did not result in activity (<0.1 nkat mg^−1^) with any of the aldose sugars. A C-terminally histidine-tagged protein was expressed from the same plasmid and in the same yeast strain. The resulting strain showed the same activity in the crude cell extract with d-xylose and NADP^+^ as the strain expressing the nontagged protein, indicating that this histidine-tag did not markedly affect the activity. The histidine-tagged protein was purified and used for a more detailed characterization. The purification resulted in a protein preparation that gave a single protein band in an sodium dodecyl sulphate-polyacrylamide gel electrophoresis with an estimated molecular mass of 50 kDa (not shown). For the tagged and purified protein the following kinetic constants were estimated. The *K*_m_ for NADP^+^ was 250±90 μM and the *V*_max_ 510±20 nkat mg^−1^ and for d-xylose the *K*_m_ was 43±5 mM and the *V*_max_ 500±30 nkat mg^−1^ (Fig. 1). The purified d-xylose dehydrogenase showed, besides the activity with d-xylose, also activity with the sugars d-glucose, l-arabinose, d-galactose, d-ribose and d-arabinose when the sugar concentration was 100 mM, but not with other sugars. This is summarized in Table 1. For the sugars which showed activity the kinetic parameters *K*_m_ and *V*_max_ were also estimated that, using the purified and histidine tagged enzyme. This is summarized in the Table 2. The highest *V*_max_ was observed with d-xylose. The highest affinity was observed with d-glucose; however, d-glucose showed a much lower *V*_max_ than d-xylose. With all the other sugars lower affinities (higher *K*_m_) and lower activities (lower *V*_max_) were observed. Leaving out the MgCl_2_ from the reaction buffer and adding EDTA did not change the enzyme activity, indicating no requirement for bivalent metal cations. To identify the product of the reaction with d-xylose the reaction mixture was analysed using HPLC. A peak with the same retention time as d-xylonic acid was identified, which was synthesized as described in ‘Materials and methods’ section. The reaction product of the enzyme reaction may actually be d-xylonate-γ-lactone, which probably is hydrolysed to d-xylonate in the alkaline conditions of the HPLC.

**Table 2:** The kinetic properties of the histidine-tagged and purified XYD1 with respect to different sugars: the NADP^+^ concentration was 0.8 mM

	*V*_max_ (nkat mg^−1^)	s^−1^	*K*_m_ (mM)	Efficacy (*V*_max_/*K*_m_) (s^−1^ M^−1^)
d-Xylose	500	21.8	43	510
d-Glucose	130	5.7	24	240
l-Arabinose	117	5.1	60	85
d-Galactose	205	8.9	100	89
d-Ribose	141	6.2	145	43
d-Arabinose	100	4.4	390	11

The other conditions are specified in ‘Materials and methods’. The activities (*V*_max_) are given in nkat mg^−1^ and in s^−1^. The affinities (*K*_m_) are given in mM and the enzyme efficacies (*V*_max_/*K*_m_) are given in s^−1^ M^−1^.

**Table 1:** Specificity of the histidine-tagged and purified d-xylose dehydrogenase

Substrate	Relative activity (%)
d-Xylose	100
d-Glucose	25
l-Arabinose	16
d-Galactose	16
d-Ribose	11
d-Arabinose	5
d-Mannose	<1
l-Rhamnose	<1
d-Lyxose	<1
d-Fructose	<1
d-Glyceraldehyde	<1
dl-Glyceraldehyde	<1

The sugar concentration was 100 mM, NADP^+^ 1 mM and the pH 8.0.

It was tested whether d-xylonic acid can be a sole carbon source for growth of *H. jecorina*. For that purpose spores of the *H. jecorina* strain VTT-D-80133 were cultivated in a liquid medium with 4% magnesium d-xylonate as a sole added carbon source. The magnesium salt was chosen because it was better soluble than the calcium d-xylonate. After 5 days of growth the dry mass was 0.1 g L^−1^. A dry mass of 0.1 g L^−1^ was also obtained when the d-xylonate was omitted indicating that d-xylonate was not used for growth. The obtained dry mass was apparently due to growth on peptone, which is a supplement in the growth medium.

## Discussion

The most common pathway in eukaryotic microorganisms for d-xylose catabolism is an oxidoreductive path in which d-xylose is first reduced to xylitol by a d-xylose reductase and subsequently oxidized to d-xylulose by xylitol dehydrogenase. In *H. jecorina* deletion of the two genes *xdh1* and *lad1* coding for two enzymes with xylitol dehydrogenase activity results in a mutant strain unable to grow on d-xylose (Seiboth *et al.*, 2003). This is a strong indication that the only path for d-xylose assimilation is through xylitol. The d-xylose dehydrogenase activity probably does not constitute part of an alternative path for d-xylose assimilation. In this communication it is also shown that d-xylonic acid is not a sufficient carbon source for growth, giving further evidence that *H. jecorina* does not have an alternative assimilatory pathway for d-xylose with d-xylonic acid as an intermediate. However, the d-xylose dehydrogenase identified from *H. jecorina* was cloned from cDNA, which was obtained from mycelia grown on a mixture of several plant polysaccharides (Margolles-Clark *et al.*, 1996), indicating that it is at least sometimes expressed. A possible role might be in the regeneration of the cofactor NADP^+^ in the presence of d-xylose. Detection of the enzyme activity in cell-free extracts is complicated by the fact that a d-xylose reductase activity with a high affinity for NADPH is present. d-Xylose dehydrogenase catalysed formation of NADPH, which is normally used to detect the activity, can be masked by the NADPH consumption by d-xylose reductase, as discussed previously (Suzuki & Onishi, 1973).

A protein, XYR1, for the transcriptional regulation of genes related to d-xylose metabolism was described recently. The XYR1 binds to a GGCTAA motif arranged as an inverted repeat in the promoter region (Stricker *et al.*, 2006). Such a motif was not found in the promoter region of the *xyd1*, suggesting that the d-xylose dehydrogenase gene is not upregulated on d-xylose.

Sugar dehydrogenases are rare in eukaryotic microorganisms. There are only a few reports of such activities. In *Schizosaccharomyces pombe* an NADP^+^-utilizing d-glucose dehydrogenase was described (Tsai *et al.*, 1995). The d-gluconate is then phosphorylated to 6-phosphogluconate which is a metabolite in the pentose phosphate pathway. In various yeast species NAD^+^-utilizing l-rhamnose dehydrogenases have been described (Twerdochlib *et al.*, 1994) and in the filamentous fungus *Aspergillus niger* an NAD^+^-dependent d-galactose dehydrogenase (Elshafei & Abdel-Fatah, 2001). Pathways similar to the Entner–Doudoroff pathway have been described where l-rhamnoate and d-galactonate were metabolized, i.e. a dehydratase converts the sugar acid to the 2-keto-3-deoxy sugar acid which is further converted by an aldolase to pyruvate and an aldehyde (Twerdochlib *et al.*, 1994; Elshafei & Abdel-Fatah, 2001). In *S. cerevisiae* an NADP^+^-utilizing d-arabinose dehydrogenase was described (Kim *et al.*, 1998). The reaction product of this enzyme is converted to d-erythroascorbic acid, a compound with chemical properties similar to l-ascorbic acid and which is not further catabolized.

In some prokaryotic organisms d-xylose is catabolized using d-xylose dehydrogenase with d-xylonate as an intermediate. The d-xylose dehydrogenase of the filamentous fungus *H. jecorina* described in this communication is probably not part of such a pathway. The role of this enzyme is still unclear.

## Statement

OnlineOpen articles are made available in accordance with the terms of the Creative Commons Deed, Attribution 2.5 (further details available from http://www.creativecommons.org), which allows Open Access dissemination of the article, but does not permit commercial exploitation or the creation of derivative works.
